# Accelerating orthodontic tooth movement: A new, minimally-invasive corticotomy technique using a 3D-printed surgical template

**DOI:** 10.4317/medoral.21082

**Published:** 2016-03-31

**Authors:** Michele Cassetta, Matteo Giansanti

**Affiliations:** 1 DDS, PhD. Department of Oral and Maxillofacial Sciences, School of Dentistry, “Sapienza” University of Rome, Italy; 2 DDS, Ph Student. Department of Oral and Maxillofacial Sciences, School of Dentistry, “Sapienza” University of Rome, Italy

## Abstract

**Background:**

A reduction in orthodontic treatment time can be attained using corticotomies. The aggressive nature of corticotomy due to the elevation of muco-periosteal flaps and to the duration of the surgery raised reluctance for its employ among patients and dental community. This study aims to provide detailed information on the design and manufacture of a 3D-printed CAD-CAM (computer-aided design and computer-aided manufacturing) surgical guide which can aid the clinician in achieving a minimally-invasive, flapless corticotomy.

**Material and Methods:**

An impression of dental arches was created; the models were digitally-acquired using a 3D scanner and saved as STereoLithography ( STL ) files. The patient underwent cone beam computed tomography (CBCT): images of jaws and teeth were transformed into 3D models and saved as an STL file. An acrylic template with the design of a surgical guide was manufactured and scanned. The STLs of jaws, scanned casts, and acrylic templates were matched. 3D modeling software allowed the view of the 3D models from different perspectives and planes with perfect rendering. The 3D model of the acrylic template was transformed into a surgical guide with slots designed to guide, at first, a scalpel blade and then a piezoelectric cutting insert. The 3D STL model of the surgical guide was printed.

**Results:**

This procedure allowed the manufacturing of a 3D-printed CAD/CAM surgical guide, which overcomes the disadvantages of the corticotomy, removing the need for flap elevation. No discomfort, early surgical complications or unexpected events were observed.

**Conclusions:**

The effectiveness of this minimally-invasive surgical technique can offer the clinician a valid alternative to other methods currently in use.

**Key words:**Corticotomy, orthodontics, CAD/CAM, minimally invasive, surgical template, 3D printer.

## Introduction

The goal of orthodontic treatment is to improve the patient’s life through the enhancement of dentofacial functions and esthetics. Orthodontic treatment of adolescent or adult patients can be challenging, often these patients request short treatments (1). Rapid orthodontic tooth movement with concomitant reduction in treatment time can be attained through a combination of orthodontic treatment and surgical alveolar corticotomies (2-6). Corticotomy is defined as any intentional surgical injury to cortical bone; it has been claimed that this technique can dramatically reduce treatment time as resistance of the dense cortical bone to orthodontic tooth movement is eliminated (2,4). Despite the different techniques described in the literature, corticotomy is the only effective and safe means of accelerating orthodontic tooth movement (7). It has been suggested that the biological basis of accelerated orthodontic tooth movement is mediated by a regional acceleratory phenomenon (8). It has also been hypothesized that corticotomy can lead to intensified osteoclastic activity resulting in osteopenia and increased bone remodeling (8). Corticotomy used to accelerate orthodontic tooth movement - also defined as corticotomy-assisted orthodontic treatment (CAOT) (7) - consists of small perforations to the alveolar bones along the route by which the tooth would move. In contrast to an osteotomy, with corticotomy cuts are made only to the cortical alveolar bone; the trabecular bone is left intact (9). Orthodontic force is applied shortly after surgery to produce the desired tooth movement and optimal bone remodeling. It has been claimed that orthodontic treatment progresses faster, and that the results are more stable after a corticotomy, with minimal risk or complications (2). Although effective, CAOT presents significant postoperative discomfort (10). The aggressive nature of this particular method due to the elevation of muco-periosteal flaps and to the duration of the surgery - has raised reluctance for its employ among both the patient and dental community (11). Initially, the cortical incisions were performed using a bone bur which could potentially damage the roots of neighboring teeth; more recently however, corticotomy has been performed by means of a piezosurgical micro-saw (12,13). The use of piezoelectric instruments appears to have several advantages such as a reduction of intraoperative bleeding and surgical trauma, and improved intraoperative visibility (14). To overcome the disadvantages of corticotomy i.e. removing the need for flaps elevation, an innovative, minimally-invasive, flapless procedure combining piezosurgical cortical micro-incisions with the use of a 3D-printed CAD-CAM (computer-aided design and computer-aided manufacturing) surgical guide has been previously described (15).

The aim of the present study is to provide detailed information on the design and manufacturing process of this 3D-printed CAD-CAM surgical guide.

## Material and Methods

**Figure 1:**
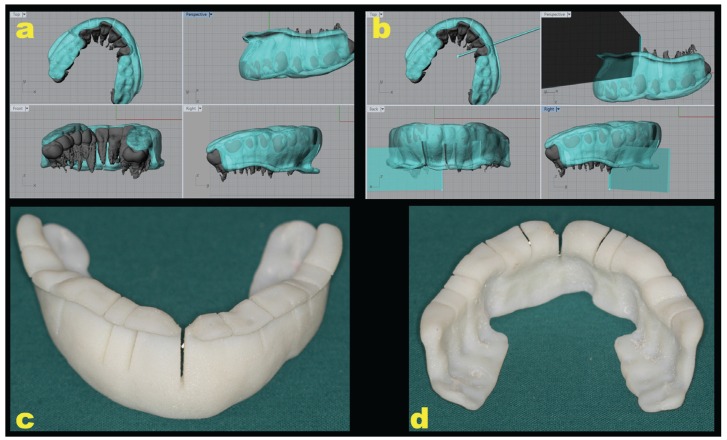
a: the STL files of template and 3D bone model acquired in Rhinoceros 3D. b: the evaluation of the space between the roots of each tooth, from upper right second molar to upper left second molar, and the design of a longitudinal axis parallel to each root. Construction and subtraction of a parallelepiped from the 3D model of the template using Rhinoceros 3D. These parallelepipeds correspond to the surgical guide slots. c, d: the 3D STL model transformed into a surgical guide using a 3D printer; the slots were designed to guide first the scalpel blade and then the piezoelectric cutting insert.

- Surgical template manufacturing:

Firstly, a preliminary impression of the dental arches, extended as far as possible into the vestibular fornix, was made. An impression tray was manufactured, a second impression was then made, a cast poured and the model digitally acquired through a 3D scanner (Easy Optical 3D Scanner, Open Technologies, Rezzato, BS, Italy). An acrylic template which covered the occlusal surface of molars and premolars and the margins of canines and incisors, extending as deep as possible in the vestibular fornix between the upper right second molar and the upper left second molar, was manufactured and scanned as well. Both the cast and the template scanner images were saved as STereoLithography (STL) files and stored. The patient underwent cone beam computed tomography (CBCT); DICOM (Digital Imaging and Communications in Medicine) images were acquired through software (Mimics, Materialise, Leuven, Belgium) which allows for the segmentation of 3D medical images; images of the jaws and teeth were transformed into 3D models and saved as STL files. With the same software, the STL of the jaw, cast, and the acrylic template were matched with point-to-point registration. The STL files of the template and jaw were acquired through a 3D modeling software application (Rhinoceros 3D, Seattle, Washington, USA). This software permits a view of the 3D model from different perspectives and planes with perfect rendering (Fig. 1a). The space between the roots of each tooth, from right second molar to left second molar, was evaluated and a longitudinal axis parallel to each root was drawn (Fig. 1b). Following the direction of this pre-determined longitudinal axis, a parallelepiped was designed and its form subtracted from the 3D model of the template in the software, creating slots of the same dimension (1mm) of the piezoelectric cutting insert (Fig. 1b). These slots were designed to be 2mm from the papilla up to the vestibular fornix and 2mm above the apex of the teeth. The 3D model of the acrylic template was thus transformed into a surgical guide with the slots designed to guide the scalpel blade first and then the piezoelectric cutting insert. The 3D STL model of the surgical guide was printed using Objet30 OrthoDesk, a 3D Printer for medical devices (Stratasys, Eden Prairie, MN, USA) (Fig. 1c,d).

**Figure 2:**
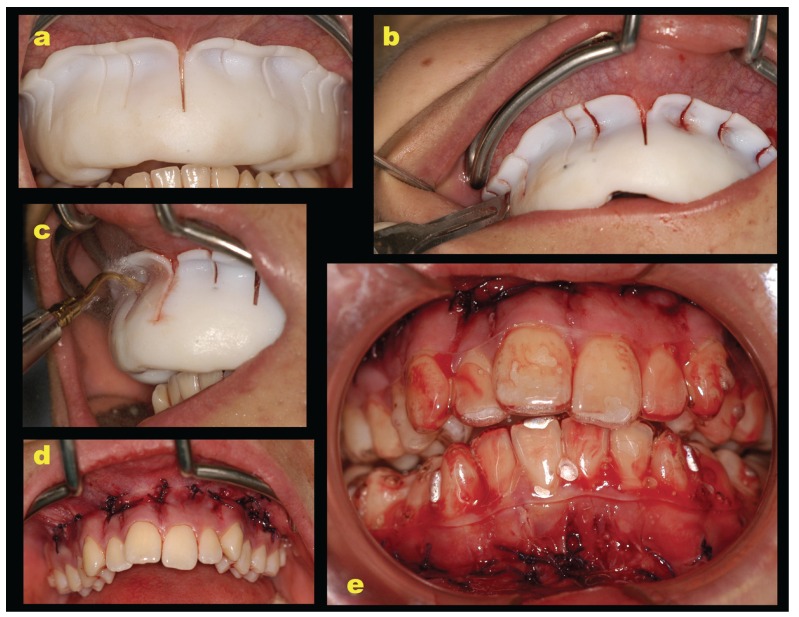
a: the positioning of 3D-printed surgical template to perform flapless corticotomy in the upper arch. The initial surgical guide stability check. b: the gingival vertical incisions using a blade number 15.c: the corticotomy vertical cuts performed using a piezosurgical device. d: the suturing of the vertical incisions. e: the positioning of clear aligners immediately after surgery.

- Surgical phase:

A mouthwash containing 0.2% chlorhexidine was used preoperatively for 1 minute. Plexus anesthesia was obtained using carbocaine 2% with adrenaline 1:100,000 as a local anesthetic. To avoid the reflection of a full-thickness flap the described surgical guide was used. After positioning the surgical guide, its stability was checked: the patient was invited to bite. The guide presented an extension on the occlusal surface of the teeth which allowed for its further stabilization in the maximum intercuspation (Fig. 2a). Gingival vertical incisions were made interproximally below the interdental papilla using a blade number 15 (Fig. 2b). The incisions crossed the periosteum allowing the blade to come into contact with the alveolar bone. The corticotomy cuts were performed through gingival incisions 2mm beyond the apices of teeth. Interproximal corticotomy cuts were extended through the entire thickness of the cortical layer, just barely penetrating into medullary bone (Fig. 2c). The design of the vertical cuts was aimed at maximizing marrow penetration and bleeding. The corticotomy was performed using a piezosurgical device to make the vertical cuts. The procedure was then completed by suturing the vertical incisions using Vicryl 3.0 thread (Vicryl Ethicon, Johnson & Johnson, Somerville, NJ, USA) (Fig. 2d). The orthodontic treatment began the same day using clear aligners (Smiletech®, Ortodontica Italia, Rome, Italy) (Fig. 2e). This clinical investigation was conducted in accordance with the ethical principles of the World Medical Association Declaration of Helsinki and was undertaken after informing the patient of the content, risks, and benefits of the study. Written consent was obtained from each participant. The investigation was reviewed and approved by the local ethics committee (Umberto I Policlinico di Roma ‘‘Corticotomia minimamente invasiva in ortodonzia, studio clinico randomizzato controllato’’ (Rif. 3730).

## Results

Using this 3D-printed CAD-CAM surgical guide no surgical complications or unexpected events, such as the presence of root damage caused by corticotomy, were observed. The above-described, minimally-invasive, flapless corticotomy procedure did not result in significant patient discomfort. This means that by using minimally-invasive surgery it is possible to overcome the disadvantages of corticotomy.

## Discussion

CAOT involves selective alveolar decortications in the form of lines and dots performed around the teeth that are to be moved. It is done to induce a state of increased tissue turnover and a transient osteopenia, which is followed by a faster rate of orthodontic tooth movement (16). To date, several different modalities have been reported which accelerate orthodontic tooth movement, including low-level laser therapy, pulsed electromagnetic fields, electrical currents, distraction osteogenesis, and mechanical vibration, but only corticotomy has shown consistent results in accelerating the orthodontic tooth movement (7).

Corticotomy cuts can be made using conventional rotatory osteotomy or piezoelectric surgery. Piezosurgery is a recently developed system for cutting bone with microvibrations which uses low frequency ultrasonic waves (24.7-29.5 kHz); the machine is programmed in accordance with the density of the bone and works only on mineralized hard tissue, not on soft tissue. During the osteotomy, the use of this ultrasonic technique minimizes the risk of accidental damage to soft tissues (10). Using the described technique, the osteotomy cut was performed without raising a mucoperiosteal flap. The piezosurgical micro-saw comes into contact with the soft tissue without causing damage. However, piezoelectric surgery is characterized by a lower cutting speed and consequently with longer surgical time (17). Using the above-described, minimally-invasive technique it is possible to reduce the treatment time thus overcoming the disadvantages of corticotomy and the longer surgical times of piezoelectric surgery.

While the patient treated with corticotomy should present a stable periodontium without periodontal disease, the present technique seems particularly indicated in adults with gingival recessions and a thin gingival biotype (8,11). The present flapless procedure combining piezosurgical cortical micro-incisions with the use of a 3D-printed CAD-CAM surgical guide do not interfere with the marginal periodontium, involves significantly less trauma to periodontal tissues and do not require hard or soft tissue grafting.

The use of ultrasonic techniques has advantages over other conventional instruments, including: A highly precise cut geometry without the need for excessive force; an efficient bone ablation, minimal risk of accidental damage to soft tissues and therefore a decrease in the risk of nerve damage (10,13,17).

An increased risk of the piezoelectric cutting insert overheating is, however, present in the described technique. Just as in computer-aided implantology, the surgical template may obstruct the correct irrigation of the piezoelectric cutting insert (18). To counteract this, it is necessary to reduce the pressure on the piezoelectric cutting insert, taking care to frequently remove the micro-saw from the surgical guide to allow the outflow of the bone fragments and cooling.

In conclusion, all the corticotomy techniques described to date are characterized by high morbidity and by possible damage to the periodontal tissues. The limited postoperative discomfort and the effectiveness of the present minimally-invasive corticotomy surgical technique should encourage the clinician to consider the use of corticotomy as a valid alternative to other techniques.
